# Variability Assessment of Aromatic and Fine Rice Germplasm in Bangladesh Based on Quantitative Traits

**DOI:** 10.1155/2016/2796720

**Published:** 2016-03-31

**Authors:** M. Z. Islam, M. Khalequzzaman, M. K. Bashar, N. A. Ivy, M. M. Haque, M. A. K. Mian

**Affiliations:** ^1^Genetic Resources and Seed Division, Bangladesh Rice Research Institute (BRRI), Gazipur 1701, Bangladesh; ^2^CIAT, HarvestPlus, Banani, Dhaka 1213, Bangladesh; ^3^Bangabandhu Sheikh Mujibur Rahman Agricultural University (BSMRAU), Gazipur 1706, Bangladesh

## Abstract

The study was conducted to investigate genetic variability among 113 aromatic and fine local rice genotypes of which five were exotic in origin. The test genotypes were evaluated for 19 growth traits, yield components, and yield. All the quantitative traits varied significantly among the test genotypes. High heritability along with high genetic advance was observed for flag leaf area, secondary branches per panicle, filled grains per panicle, grain length, grain breadth, grain length breadth ratio, and 1000 grain weight. Such findings suggested preponderance of additive gene action in gene expression for these characters. Grain yield was significantly and positively correlated with days to flowering, days to maturity, panicle length, filled grains per panicle, and 1000 grain weight. According to *D* ^2^ cluster analysis, 113 test genotypes formed 10 clusters. Selection of parents from the clusters V and X followed by hybridization would possibly result in desirable heterosis for the development of heterotic rice hybrids. Finally, molecular characterizations of the studied germplasm are required for high resolution QTL mapping and validating the presence of candidate genes responsible for valuable characters.

## 1. Introduction

Bangladesh is mainly a country of rice based cropping system, where thousands of local rice varieties are being cultivated from the time immemorial [1]. To date, farmers across the country are used to cultivate different local varieties or landraces particularly in the unfavourable ecosystems. Local variety including aromatic rice genotypes occupied about 12.16% of the rice growing areas in Bangladesh [2]. Many of these local varieties have some special characteristics such as aroma, better taste, and higher cooking quality which also provide additional value in socioeconomic aspects. Moreover, aromatic rice constitutes a special group of rice genotypes well known in many countries across the world for their aroma and/or super fine grain quality [3]. Bangladesh has a stock of above 8,000 rice germplasms of which nearly 100 are aromatic [1, 4]. It is worthwhile to mention that aromatic rice is closely related to the social and cultural heritage in Bangladesh and it is consumed during weddings and other festivals [5]. Aromatic and fine rice germplasm native to Bangladesh generally have short bold and medium bold grain type with mild to strong aroma [6, 7]. In Bangladesh, among the different aromatic rice varieties, Chinigura is the predominant one that covers more than 70% of rice farms in the northern districts of Naogaon and Dinajpur. Other important aromatic rice varieties are Kalijira (predominantly grown in Mymensingh) and Kataribhog (mainly cultivated in Dinajpur) [8]. Most of the aromatic rice varieties in Bangladesh are of locally adapted, photoperiod-sensitive, and grown during Aman season under rainfed lowland ecosystem. The production cost of aromatic and fine rice is low compared to that of coarse rice. Therefore, the income potential is higher with aromatic fine rice cultivation, since its cultivation does not usually require additional expenditures on fertilizer, pesticides, and irrigation. However, the average yield of high yielding rainfed lowland rice is 3.4 t/ha, whereas that of aromatic rice is 2.0–2.3 t/ha [9].

Knowledge on genetic diversity among crop populations and its quantitative assessment usually helps a breeder to select suitable parents to be utilized in breeding programmes [10–15]. Among the different cereal crops, rice is (*Oryza sativa*) one of the best models to undertake the study of genome structure and genetic diversity. Its diploid genome is relatively smaller in size (430 Mb) with a significant level of genetic polymorphism and a large amount of well-conserved genetically diverse material [16–18].

In a breeding programme, genetic improvement primarily depends upon the amount of genetic variability present in the population. In many cases, characters are mostly governed by poly genes which are highly influenced by the environment. Therefore, it is difficult to predict whether the existing variability is heritable or not. Furthermore, heritability of a genetic trait is very important in determining the response to selection because it implies the extent of transmissibility of that trait into next generations [19, 20]. In addition, high genetic advance coupled with high heritability offers the most effective condition for selection for a specific character [21].

These days, plant breeders usually evaluate genetic diversity on the basis of morphological traits because they are more economic, faster, and easier to score compared to the molecular traits [22, 23]. Investigation to these traits also does not require any sophisticated procedure or advanced equipment. In addition, these traits can be transmitted without adapting any special biochemical or molecular techniques. The rice plant is morphologically diverse, especially in terms of the vegetative traits such as plant height and leaf length. Our previous studies involving local aromatic and nonaromatic rice germplasm from Bangladesh using morphological, physicochemical, and molecular markers revealed high genetic diversity [6, 24–28]. However, such investigations on aromatic and fine rice genotypes are not yet to be conducted. Therefore, the present study was undertaken to assess the genetic diversity of aromatic and fine rice genotypes in Bangladesh.

## 2. Materials and Methods

### 2.1. Experimental Site

The experiment was conducted at the research farm of Bangladesh Rice Research Institute (BRRI), Gazipur, during July to December (T. Aman season), 2011. Geographically, the place is located at about 24.00°N latitude and 90.25°E longitude with an elevation of 8.4 meters from the sea level and is characterized by subtropical climate. The soil of the experimental site was clay loam in texture.

### 2.2. Plant Materials

A total of 113 aromatic and fine rice genotypes (Table 1) were selected from BRRI genebank. Pregerminated seeds were sown in the seed bed.

### 2.3. Experimental Design and Setting the Experiment

The experiment was conducted following a randomized complete block design with three replicates for each treatment. Thirty-day-old seedlings of each test genotypes were transplanted on the 15th August, 2011 using single seedling per hill in 2.4 m^2^ plot with 25 cm and 20 cm space between rows and plants, respectively.

### 2.4. Intercultural Operations

Fertilizers were applied @ 80 : 60 : 40: 12 kg N : P : K : S per hectare. However, except N, the other fertilizers were applied at final land preparation. Nitrogen was applied in three equal splits, at 15 days after transplanting (DAT), at 35 DAT, and just before flowering. Intercultural operations and pest control measures were done as and when necessary.

### 2.5. Data Collection

Data were collected on culm diameter (mm), flag leaf area (cm^2^), plant height (cm), days to flowering, days to maturity, effective tiller number (ET No.), panicle length (cm), primary branch length (cm), secondary branch length (cm), primary braches per panicle, secondary branches per panicle, number of filled grains per panicle, number of unfilled grains per panicle, grain length (mm), grain breadth (mm), grain length breadth ratio, 1000-grain weight (g), grain yield per hill (g), and harvest index (HI). Kernel quality was determined using dehusked grains. Kernels were classified on the basis of length (size) and for *L*/*B* ratio (shape) following classification described by Cruz and Khush [29] (Table 2).

### 2.6. Aroma Test

Aroma was detected by sniffing and was scored as nonscented, lightly scented, and scented following 1.7% KOH based method [30] (Table 2).

### 2.7. Statistical Analysis

Univariate analysis of the individual character (ANOVA) including the estimation of mean, range, and coefficient of variation (CV%) was conducted using a statistical software package MstatC. The test of significance was performed using Fisher's (*F*) test. Multivariate analysis was conducted using another statistical software package GENSTAT version 5.5. Genetic parameters were also estimated to understand genetic variations among the test genotypes and to determine genetic and environmental effects on different characters. These genetic parameters were calculated using the following formula [31–33]. These parameters include the following:

(a) Genotypic variance, *σ*_*g*_^2^ = (GMS − EMS)/*r*,
 where GMS is the genotypic mean sum of squares, EMS is the error mean sum of squares, and *r* is number of replication.
(b) Phenotypic variance, *σ*_*p*_^2^ = *σ*_*g*_^2^ + *σ*_*e*_^2^,
 where *σ*_*g*_^2^ is the genotypic variance and *σ*_*e*_^2^ is the mean squares of error.
(c) Genotypic coefficient of variation $\left(GCV\right)\%=\sqrt{\sigma_{g}^{2}}/\overset{-}{x}\times 100$,
 where *σ*_*g*_^2^ is the genotypic variance and $\overset{-}{x}$ is the mean of character.
(d) Phenotypic coefficient of variation $(PCV)\%=\sqrt{\sigma_{p}^{2}}/\overset{-}{x}\times 100$,
 where *σ*_*p*_^2^ is the phenotypic variance and $\overset{-}{x}$ is the mean of trait.
(e) Heritability (broad sense) *h*_*B*_^2^% = *σ*_*g*_^2^/*σ*_*p*_^2^ × 100,
 where *σ*_*g*_^2^ is the genotypic variance and *σ*_*p*_^2^ is the phenotypic variance.
(f) Expected genetic advance (GA): GA(%) = *K* × *σ*_*p*_^2^ × *h*_*B*_^2^ × 100,
 where GA is a percent of the mean assuming selection of the superior 5% of accession: $GA(\%)=K\times \sqrt{\sigma_{p}^{2}}/\overset{-}{x}\times h_{B}^{2}\times 100$,
 where *K* is a constant, $\sqrt{\sigma_{p}^{2}}/\overset{-}{x}$ is the phenotypic standard deviation, *h*_*B*_^2^ is the heritability, and $\overset{-}{x}$ is the mean of traits.

## 3. Result

### 3.1. Variation in Grain Diversity and Genetic Parameters among Accessions

**Figure 1:**
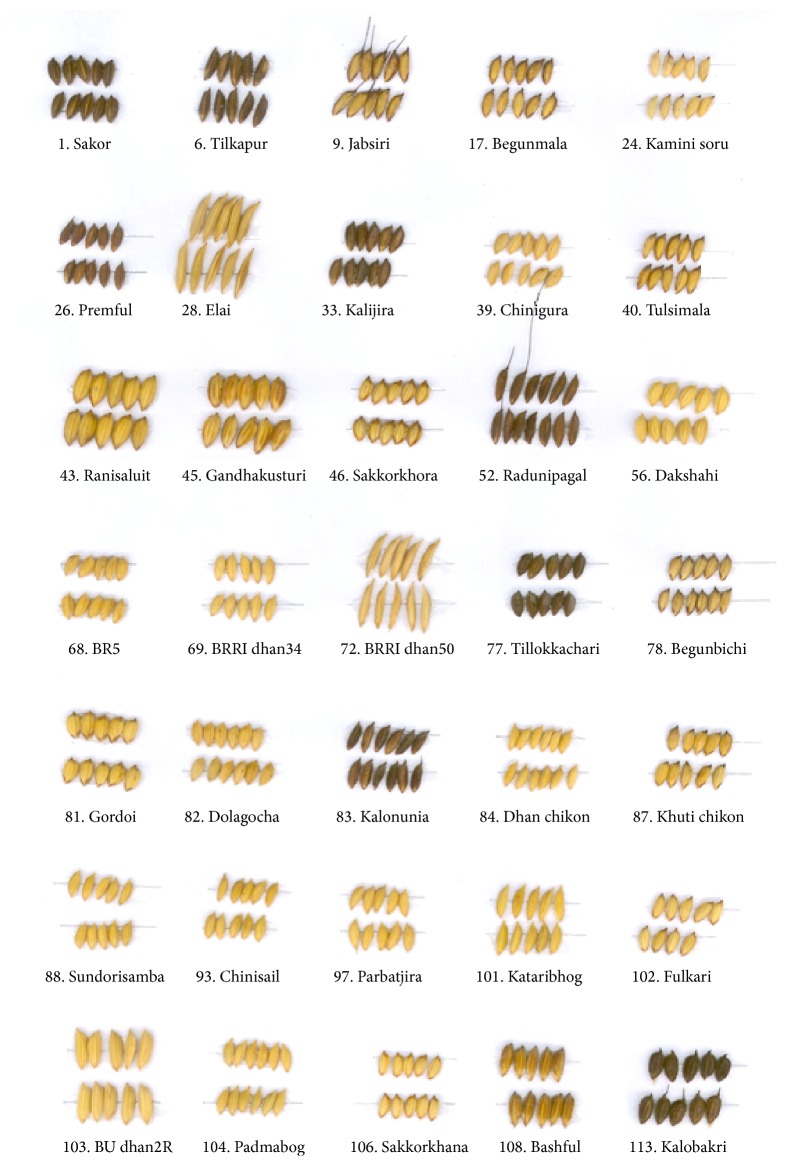
Variation in grain morphology of some aromatic and fine rice genotypes.

The grain morphology varied considerably in genotypes collected from BRRI genebank (Figure 1) with respect to awning, colour and size of awns, lemma and palea with presence or absence of coloured furrows and spots, pubescence, and varied coloured apiculus and sterile lemma. Analysis of variance of 19 quantitative characters based on individual sample means showed highly significant (*P* ≤ 0.01) variations among the genotypes for all the characters outlined in Table 3. The range, mean, standard error, coefficients of variation, and *F* value of 19 characters are presented in Table 4. The coefficient of variation ranged from 1.69 to 35.81% which indicates considerable variation among the character studied. Out of 19 traits, unfilled grains per panicle, harvest index, yield per plant, filled grains per panicle, primary branches per panicle, and secondary branches per panicle found with relatively higher coefficient of variation (35.81, 20.45, 18.70, 16.79, 11.61, and 11.03%, resp.) than the other traits. These possibly occurred because of sampling error and/or characters were more influenced by the environmental factors. In this study, most of the growth traits showed higher PCV compared to yield and yield component traits. However, lower PCV belonged to days to maturity (5.79%) while unfilled grains per panicle (46.57%) were recorded with higher value. Secondary branches per panicle (34.95%), 1000 grain weight (34.20%), and filled grains per panicle (29.32%) were recorded with higher values of PCV. However, panicle length (6.31%), days to flowering (7.40%), and plant height (10.04%) were found with lower values. The higher GCV was associated with 1000-grain weight (33.18%) whereas the value was fairly low in case of panicle length (5.06%). Results also showed narrow differences between PCV and GCV for most of the traits. Heritability ranged from 29.03 to 97.44%. The highest and the lowest amount of heritability were recorded at grain length and yield per plant, respectively.

Days to flowering, grain breadth, grain length breadth ratio, plant height, and days to maturity were highly heritable, all with an estimated *H* ^2^ > 0.90 whereas other characters showed relatively low heritability. GA ranged from 0.03% for harvest index to 48.19% for filled grains per panicle. The genetic advance as percent of mean (GA%) ranged from 6.41% in panicle length to 50.85% in 1000 grain weight. In this study, flag leaf area, secondary branches per panicle, filled grains per panicle, grain length, grain breadth, grain length breadth ratio, and 1000-grain weight showed high heritability and high genetic advance indicated the presence of additive genes controlling these characters (Table 5).

### 3.2. Association between Traits

Pearson's correlation coefficient was computed between 19 quantitative traits among 113 accessions of aromatic and fine rice genotypes (Table 6). Culm diameter was significantly and positively correlated with flag leaf area, days to flowering, days to maturity, plan height, and primary branches per panicle. Plant height showed highly significant positive correlation with culm diameter, days to flowering, days to maturity, and panicle length. Grain yield was highly significant (*P* < 0.01) and positively correlated with days to flowering (*r* = 0.407), days to maturity (*r* = 0.431), filled grains per panicle (*r* = 0.267), primary branch length (*r* = 0.324), secondary branch length (*r* = 0.324), and 1000-grain weight (*r* = 0.258) and positively correlated with panicle length (*r* = 0.190) and secondary branches per panicle (*r* = 0.231).

### 3.3. Principal Component Analysis (PCA)

Eigen values (latent roots) of 19 principal component axes and percentage of total variation accounted for them obtained from component analysis are presented in Table 7. The result revealed that the first axis largely accounted for the variations observed among the genotypes (48.8%) followed by the second axis (10.37%). The first nine axes accounted for about 90% of the total variations among the 19 characters describing 113 aromatic and fine rice genotypes where only 59.17% variation was accounted for the first two axes.

### 3.4. Cluster Analysis

The pattern of distribution of 113 aromatic and fine rice genotypes were grouped into 10 clusters shown in Table 8. The number of genotypes ranged from 3 to 19 in different cluster. The distribution pattern indicated that the maximum number of test genotypes (19) was grouped into the cluster I followed by 18 in clusters VIII, 17 in clusters III, 13 in clusters IV, 11 in clusters II, 10 in clusters X, 8 in clusters V and IX, and 6 in cluster VII. Cluster VI contained the lowest (3) number of genotypes.

Results of 10 higher and 10 lower intergenotypic distances estimated from distant matrix of Principal Coordinate Analysis are shown in Table 9. Highest intergenotypic distance was 2.274 observed between Gopalbhog and Kalobakri followed by the distance of 2.126 observed between Haitsail TAPL101 and Kalobakri. The 10th highest distance of 1.522 was observed between Jirabuti and Straw TAPL554 followed by 1.528 observed between Ranisaluit and Jirakatari. The lowest distance was calculated (0.299) between Doiagura and Jiradhan followed by the distance of 0.301 observed between Kamini soru and Jirabhog (bolder).

Intra- and intercluster distances value are presented in Table 10. There were marked variations in intracluster distances which ranged from 0.61 in cluster VI to 1.27 in cluster II indicating homogeneous nature of the genotypes within the cluster. The highest intracluster distance was computed for cluster II (1.27) which was comprised of eleven genotypes followed by cluster IV (1.01) with thirteen genotypes. The genotypes under cluster II (with the highest intracluster mean) were most heterogeneous and genotypes under cluster VI (with the lowest intracluster mean) were comparatively homogenous.

The intercluster distances ranged from 3.710 to 16.116. Regarding the intercluster distance, the highest value was found between clusters V and X (16.116) followed by clusters II and X (15.791) and so on. On the other hand, the lowest intercluster distance was observed between clusters I and III (3.710) followed by clusters III and IX (3.775) indicating that genotypes of these clusters were genetically closed.

The mean values for all of 19 characters along with the marking of the highest (*H*) and lowest (*L*) for each of the cluster are presented in Table 11. Differences in cluster means existed for almost all the characters. Genotypes of cluster VI produced the highest mean for days to flowering (DF), days to maturity (DM), plant height (PH), and yield per plant (Y/P). Genotypes in cluster II had higher mean values for flag leaf area (FLA), secondary branch length (SBL), grain length (GL), grain length breadth ratio (GLBR), and 1000-grain weight (1000). Higher mean values for panicle length (PL), secondary branches per panicle (SB No.), filled grains per panicle (FG/P), and harvest index (HI) were recorded in cluster X whereas those for effective tiller (ET No.) per plant and grain breadth (GB) were recorded in cluster IV.

## 4. Discussion

During the current study, all traits showed highly significant (*P* < 0.01) variations among 113 accessions, which originated in Bangladesh except Khazar, Basmati 37, Basmati 370, Basmati Sufaid 106, and Basmati Sufaid 187 genotypes. Our results are in close agreement with those of Pandey et al. [34] who recorded highly significant variability among the different rice genotypes. Similarly the finding of Wang et al. [35] also gives support to the current findings. The findings of Chandra et al. [36] and Abarshahr et al. [37] further strengthen the current findings, who also found valuable and highly significant and positive variability among their studied genotypes.

The dependence of grain yield on other traits has been reported for many crops [38]. As mentioned, in this study, yield of plant had positive correlation with 8 quantitative traits. Lasalita-Zapico et al. [39] studied correlation coefficient of 10 quantitative traits for 32 upland rice varieties. In this distinguished significant positive correlation the majority of the morphological traits was recorded except flag leaf angle that had negative correlation with most of characters such as panicle length, leaf length, leaf width, ligule length, leaf area, and culm length. In our studies, grain yield positively correlated with panicle length. The findings indicate that plants with high panicles have high number of filled grains thereby increasing rice yield. Similar correlations were reported by Zafar et al. [40].

The calculation of heritability and genetic advance are used to help the breeder to select traits that are highly heritable as compared to a trait which is less heritable [33]. Both high heritability and genetic advance value obtained in this study, flag leaf area, secondary branches per panicle, filled grains per panicle, grain length, grain breadth, length breadth ratio, and 1000-grain weight indicated reasonable variation for this traits. This suggests that selection can be easily practiced by using these traits to improve grain yield in aromatic rice genotypes. The results support the findings of Sedeek et al. [41], Laxuman et al. [42], and Pandey et al. [34] who reported such type of heritability in rice.

In the present study, 113 aromatic and fine rice genotypes were clustered into ten groups based on 19 quantitative traits. This result supports the findings of Singh et al. [43] and Rao et al. [44] who reported ten clusters in rice genotypes. Ahmadikhah et al. [38] clustered 58 rice varieties into four groups based on 18 morphological traits and genetic distance was around 0.75. Group A was comprised of only one genotype and groups B, C, and D contained 14, 20, and 23 genotypes, respectively. Veasey et al. [45] computed clustering for 23 populations of rice by 20 morphological characteristics. So the varieties were clustered into 10 groups; the last group was the biggest group with seven members and groups 1, 2, 7, and 8 were the smallest groups including only one variety. So, genotypes having distant clusters could be hybridized to get the higher heterotic responses. The similar findings were also reported in a number of previous studies [18, 46–48].

Principal component analysis indicated diversity among 113 aromatic and fine rice genotypes. “Proper values” measure the importance and contribution of each component to total variance, whereas each coefficient of proper vectors indicates the degree of contribution of every original variable with which each principal component is associated. The higher the coefficients are, regardless of the direction (positive or negative), the more effective they will be in discriminating between accessions. In the present study, the first three axes accounted for about 66% of the total variations. Lasalita-Zapico et al. [39] computed approximately 82.7% of total variation among 32 upland rice varieties, 66.9% variation for PC1, and 15.87% for PC2. Rajiv et al. [49] reported the first two principal components accounting for 82.1% of total variation in control and 68.6% in the stress induced genotypes. To obtain greater heterosis, genotypes having distant clusters could be used as parents for hybridization program. In Bangladesh, most of the aromatic rice genotypes are traditional, photoperiod-sensitive, tall stature, and lower yields with mild to strong aroma and also they showed high variability (6, 28). In the present study, it was observed that the genotypes in clusters V and X (16.116) were more diverse than the genotypes of clusters I and III (3.710). Considering cluster distance and cluster mean, the highest mean value for panicle length (cm), secondary branch length (cm), filled grains per panicle, and harvest index was observed in cluster X, which means that those traits might be selected for their high heterosis. Therefore, selection of parents for hybridization program from clusters V and X may result in the desirable heterosis for heterotic rice hybrids. Genotypes under cluster II may also give higher heterosis, if crossing is done within the genotypes of this cluster due to high value of intracluster distance.

## 5. Conclusion

In the present study, flag leaf area, secondary branches per panicle, filled grains per panicle, grain length, grain breadth, grain length breadth ratio, and 1000-grain weight showed high heritability and high genetic advance in percent of mean had high heritability and high genetic advance. Yield of plant had positively correlated with days to flowering, days to maturity, panicle length, filled grains per panicle, and 1000-grain weight. The cluster analysis placed 113 aromatic and fine rice genotypes into ten groups. The highest intercluster distance was observed between clusters V and X followed by clusters II and X. The maximum value of intercluster distance indicated that the genotypes belonging to cluster V were far diverged from those of cluster X. So, it is expected in our results that parent's selection for hybridization from the clusters V and X may give the desirable heterosis for heterotic rice hybrids. Finally, molecular characterizations of the studied germplasm are required for high resolution QTL mapping and validating the presence of candidate genes responsible for valuable characters.

## Figures and Tables

**Table 1:** Information on place of collection, source, and local name of the aromatic and fine rice accessions.

Sl. No.	Genotypes	Acc. No./source	Place of collection	Kernel size and shape	1.7% KOH (aroma)
1	Sakor	197	Mymensingh	Short, bold	Lightly scented
2	Sagardana	229	Mymensingh	Short, medium	Lightly scented
3	Nunia	233	Mymensingh	Short, medium	Lightly scented
4	Chini Sagar (2)	245	Mymensingh	Short, medium	Scented
5	Meny	288	Gaibandha	Short, bold	Scented
6	Tilkapur	296	Gaibandha	Short, medium	Lightly scented
7	Binnaphul	315	Gaibandha	Short, medium	Lightly scented
8	Kalobhog	318	Gaibandha	Short, medium	Scented
9	Jabsiri	331	Gaibandha	Short, medium	Scented
10	Kalgochi	352	Gaibandha	Short, bold	Scented
11	Chinisakkor	387	Rajshahi	Short, medium	Scented
12	Chiniatob	399	Rajshahi	Short, medium	Scented
13	Noyonmoni	461	Rajshahi	Short, medium	Scented
14	Saubail	873	Sylhet	Short, medium	Scented
15	Chinniguri	1880	Kishoreganj	Short, bold	Scented
16	Kalomala	1886	Kishoreganj	Short, medium	Scented
17	Begunmala	1896	Kishoreganj	Short, medium	Scented
18	Gopalbhog	1938	Kishoreganj	Short, medium	Scented
19	Tulsimoni	1980	Jamalpur	Short, medium	Scented
20	Jirabuti	1984	Mymensingh	Short, bold	Scented
21	Khirshabuti	1996	Tangail	Short, medium	Scented
22	Rajbut	1999	Tangail	Short, medium	Scented
23	Soru kamina	2015	Satkhira	Short, bold	Lightly scented
24	Kamini soru	2027	Satkhira	Short, bold	Lightly scented
25	Doiarguru	2037	Khulna	Short, bold	Lightly scented
26	Premful	2041	Satkhira	Short, medium	Scented
27	Begun bichi	2073	Kishoreganj	Short, bold	Lightly scented
28	Elai	2423	Dhaka	Long, slender	Nonscented
29	Gua masuri	3666	Sherpur	Short, medium	Nonscented
30	Luina	3676	Netrokona	Short, medium	Scented
31	Lal Soru	4135	Dinajpur	Short, medium	Scented
32	Chini Kanai	4356	Khulna	Short, bold	Scented
33	Kalijira (short grain)	4357	Khulna	Short, bold	Scented
34	Rajbhog	4360	Khulna	Short, medium	Scented
35	Philippines Kataribhog	4365	Dinajpur	Short, medium	Scented
36	Baoibhog	4813	Kurigram	Short, medium	Scented
37	Baoijhaki	4826	Dinajpur	Short, medium	Lightly scented
38	Jirabhog (Bolder)	4828	Dinajpur	Short, bold	Lightly scented
39	Chinigura	4867	Mymensingh	Short, bold	Scented
40	Tulsimala	4870	Mymensingh	Short, bold	Scented
41	Bashmati 370	4904	Pakistan	Medium, slender	Scented
42	Uknimodhu	5083	Rangpur	Short, medium	Scented
43	Ranisalut	5286	Khulna	Short, bold	Lightly scented
44	Jira dhan	5313	Khulna	Short, bold	Scented
45	Gandhakusturi	5319	Bagerhat	Short, bold	Nonscented
46	Sakkorkhora	5347	Barguna	Short, bold	Scented
47	Badshabhog	5349	Bagerhat	Short, bold	Scented
48	Jirakatari	5975	Dinajpur	Short, bold	Scented
49	Desikatari	5978	Dinajpur	Short, medium	Scented
50	Thakurbhog	5983	Sylhet	Short, medium	Nonscented
51	Tulsimaloty	6638	Tangail	Short, bold	Scented
52	Raduni pagal	6711	Rajshahi	Short, medium	Scented
53	Sugandhi dhan	7063	Nawabganj	Short, medium	Nonscented
54	Kalijira (long grain)	4358	Khulna	Short, medium	Scented
55	Jesso balam TAPL-25	2454	GRSD, BRRI	Short, medium	Scented
56	Dakshahi	983	Khulna	Short, bold	Nonscented
57	Hatisail TAPL-101	2528	GRSD, BRRI	Short, bold	Scented
58	Khasa	682	Comilla	Short, medium	Scented
59	Buchi	369	Gaibandha	Short, bold	Scented
60	Awned TAPL-545	2939	GRSD, BRRI	Short, bold	Scented
61	Black TAPL-554	2947	GRSD, BRRI	Short, bold	Scented
62	Straw TAPL-500	2898	GRSD, BRRI	Long, slender	Scented
63	Dubsail	4840	Satkhira	Short, bold	Scented
64	Duksail	2028	Satkhira	Short, bold	Nonscented
65	Khaskani	4341	Jessore	Short, medium	Scented
66	Khazar	4921	Iran	Long, slender	Nonscented
67	Basmati Sufaid 106	4498	Pakistan	Medium, slender	Lightly scented
68	BR5	4343	GRSD, BRRI	Short, bold	Scented
69	BRRI dhan34	7093	GRSD, BRRI	Short, medium	Scented
70	BRRI dhan37	7094	GRSD, BRRI	Short, medium	Scented
71	BRRI dhan38	7095	GRSD, BRRI	Medium, slender	Scented
72	BRRI dhan50	6882	GRSD, BRRI	Long, slender	Lightly scented
73	Khasa Mukpura	7586	Khagrachhari	Short, medium	Scented
74	Uknimodhu	298	Gaibandha	Short, bold	Scented
75	Bawaibhog-2	301	Gaibandha	Short, medium	Scented
76	Chiniatob-2	398	Rajshahi	Short, bold	Scented
77	Tilokkachari	758	Chittagong	Short, bold	Scented
78	Begunbichi-2	508	Rangpur	Short, bold	Scented
79	Chinairri	764	Chittagong	Short, bold	Scented
80	Bhatir chikon	774	Chittagong	Short, medium	Scented
81	Gordoi	1908	Kishoreganj	Short, bold	Nonscented
82	Dolagocha	451	Rajshahi	Short, bold	Nonscented
83	Kalonunia	537	Rangpur	Short, medium	Lightly scented
84	Dhan chikon	538	Dinajpur	Short, medium	Lightly scented
85	Badshabhog-2	03	Dhaka	Short, bold	Scented
86	Thakurbhog-2	872	Sylhet	Short, bold	Nonscented
87	Khuti chikon	4107	Comilla	Short, bold	Lightly scented
88	Sunduri samba	4803	Rajshahi	Short, medium	Nonscented
89	Basmati	4754	Barguna	Short, bold	Scented
90	Basmati 37	4491	India	Long, slender	Lightly scented
91	Basnatu sufaid 187	4499	Pakistan	Long, slender	Lightly scented
92	Tulsimala-2	7342	Sherpur	Short, bold	Lightly scented
93	Chinisail	7343	Sherpur	Short, medium	Lightly scented
94	Malshira	7347	Sherpur	Short, bold	Lightly scented
95	Sadagura	—	Khagrachhari	Short, medium	Lightly scented
96	Modhumadab	7352	Habiganj	Short, medium	Lightly scented
97	Parbatjira	7351	Habiganj	Short, bold	Lightly scented
98	Chinikanai-2	7350	Dinajpur	Short, bold	Lightly scented
99	Meedhan	7537	Habiganj	Short, medium	Lightly scented
100	Gobindhabhog	—	Jessore	Short, medium	Lightly scented
101	Kataribhog	7082	Dinajpur	Short, medium	Scented
102	Fulkari	7531	Habiganj	Short, bold	Lightly scented
103	BU dhan2R	7413	GRSD, BRRI	Long, slender	Lightly scented
104	Padmabhog	4812	Kurigram	Short, medium	Lightly scented
105	Dudsail	4840	Satkhira	Short, medium	Lightly scented
106	Sakkorkhana	4761	Barguna	Short, medium	Scented
107	Maloti	169	Tangail	Short, medium	Lightly scented
108	Bashful	4215	Kishoreganj	Short, medium	Scented
109	Kalijira TAPL-64	2492	GRSD, BRRI	Short, medium	Scented
110	Oval TAPL-2990	2990	GRSD, BRRI	Short, medium	Lightly scented
111	Kalijira TAPL-68	2496	GRSD, BRRI	Short, medium	Scented
112	Kalijira TAPL-74	2501	GRSD, BRRI	Short, bold	Scented
113	Kalobakri	2108	Narsingdi	Short, bold	Scented

**Table 2:** List of quantitative traits of 113 aromatic and fine rice genotypes.

Traits	Method of evaluation
Culm diameter (CD, mm)	Outer diameter internodes of the 10 culms were measured and averaged
Flag leaf area (FLA, cm^2^)	Flag leaf area (cm^2^) = flag leaf length (cm) × maximum width (cm) × 0.75
Plant height (PH, cm)	The average of height from the base to the tip of last leaf (flag leaf)
Days to flowering (DF, days)	The number of days from seeding to flowering day
Days to maturity (DM, days)	The number of days from seeding to maturing day
Effective tiller number (ET No.)	Counting of effective tiller per hill
Panicle length (PL, cm)	Distance between apex of the panicle (excluding awn) and top most node (neck node) of the culm
Primary braches per panicle (PBP, no.)	Primary branches were counted from 5 randomly selected panicles and averaged
Primary branch length (PBL, cm)	Lengths of the primary branches present in a panicle were measured (cm) from five panicles and averaged
Secondary branches per panicle (SBP, no.)	Secondary branches were counted from 5 randomly selected panicles and averaged
Secondary branch length (SBL, cm)	Length (cm) of the 30 random secondary branches from five randomly selected panicles and averaged
Filled grains per panicle (FGP, no.)	Number of filled grains per panicle was counted from 10 randomly selected panicles and averaged
Unfilled grains per panicle (UFGP, no.)	Number of unfilled grains per panicle was recorded from 10 randomly selected panicles and averaged
Grain length (GL, mm)	Length (mm) of a grain was measured by a digital slide caliper from 10 randomly selected fertile grains excluding awn and averaged
Grain breadth (GB, mm)	Breadth of a grain (mm) was measured form 10 randomly selected fertile grains by a digital slide caliper and averaged
Grain length breadth ratio (GLBR)	Dividing grain length by grain breadth and averaged
1000-grain weight (TGW, g)	200 grains were weighted then 1000-weight grains were calculated from these weights
Yield per plant (GYP, g)	Ten randomly selected plants per replication and averaged
Harvest index (HI)	Ratio of grain yield to biological yield

**(a):** 

Source of variation	df	CD (mm)	FLA (cm^3^)	DF	DM	PH (cm)	ET No.	PL (cm)	PB No.	PBL (cm)	SB No.
Replication	2	1.173	32.105	0.51	1.507	250.964	28.769	15.972	4.975	0.081	38.629
Genotype	112	1.422^*∗∗*^	155.904^*∗∗*^	170.559^*∗∗*^	164.857^*∗∗*^	615.038^*∗∗*^	5.491^*∗∗*^	7.455^*∗∗*^	5.129^*∗∗*^	3.825^*∗∗*^	365.148^*∗∗*^
Error	224	0.158	17.342	2.257	4.951	16.130	1.156	1.164	1.441	0.593	12.993
CV%		8.60	11.70	2.10	2.69	2.78	10.93	3.77	11.61	7.71	11.03

**(b):** 

Source of variation	SBL (cm)	FGP	UFGP	GL (mm)	GB (mm)	GLBR	TGW (g)	YP (g)	HI
Replication	0.044	3274.094	544.179	0.234	0.039	0.107	35.785	18.735	0.016
Genotype	0.228^*∗∗*^	4828.209^*∗∗*^	741.844^*∗∗*^	5.412	0.423^*∗∗*^	1.48^*∗∗*^	58.883^*∗∗*^	11.961^*∗∗*^	0.005^*∗∗*^
Error	0.034	675.308	241.344	0.047	0.017	0.03	1.206	1.624	0.002
CV%	6.66	16.79	35.81	3.06	5.58	5.55	8.31	18.70	20.44

*∗∗* indicates significance at 1% level of probability.

CD: culm diameter (mm), FLA: flag leaf area, DF: days to flowering, DM: days to maturity, PH: plant height, ET No.: effective tiller number, PL: panicle length, PB No.: primary branches per panicle, PBL: primary branch length, SB No.: secondary branches per panicle, SBL: secondary branch length, FGP: filled grains per panicle, UFGP: unfilled grains per panicle, GL: grain length, GB: grain breadth, GLBR: grain length breadth ratio, TGW: 1000-grain weight, Y/P: yield per plant, and HI: harvest index.

**Table 4:** Variability in different quantitative characters in 113 aromatic and fine rice genotypes.

Characters	Range	Mean	SE	CV%	*F*-value
CD (mm)	3.14–7.50	4.63	0.065	8.60	8.98^*∗∗*^
FLA (cm^3^)	21.94–54.43	35.28	0.599	11.70	8.99^*∗∗*^
DF	76.00–125	100.5	0.709	2.45	75.58^*∗∗*^
DM	102–150	126.00	0.697	1.69	33.30^*∗∗*^
PH (cm)	79.00–179.00	129.00	1.382	2.74	38.13^*∗∗*^
ET No.	4.00–13.87	9.84	0.127	10.39	4.75^*∗∗*^
PL (cm)	24.13–33.00	28.62	0.149	3.77	6.40^*∗∗*^
PB No.	6.33–13.33	10.34	0.123	11.61	3.56^*∗∗*^
PBL (cm)	8.84–14.62	10.75	0.106	7.71	6.45^*∗∗*^
SB No.	12.20–65.67	32.67	1.038	11.03	28.10^*∗∗*^
SBL (cm)	1.95–3.58	2.75	0.026	6.66	6.79^*∗∗*^
FG/P	79.00–262.00	170.50	3.774	16.79	7.15^*∗∗*^
UFG/P	4.00–74.00	39.00	1.479	35.81	3.07^*∗∗*^
GL (mm)	5.62–12.24	7.11	0.126	3.06	114.34^*∗∗*^
GB (mm)	1.68–3.60	2.33	0.035	5.58	24.95^*∗∗*^
GLBR	1.97–5.60	3.10	0.066	5.55	49.59^*∗∗*^
TGW (g)	7.70–28.33	13.22	0.417	8.31	48.81^*∗∗*^
YP (g)	6.47–17.45	12.38	0.188	18.70	2.23^*∗∗*^
HI	0.16–0.34	0.24	0.004	20.45	1.93^*∗∗*^

*∗∗* indicates significance at 1% level of probability.

CD: culm diameter (mm), FLA: flag leaf area, DF: days to flowering, DM: days to maturity, PH: plant height, ET No.: effective tiller number, PL: panicle length, PB No.: primary branches per panicle, PBL: primary branch length, SB No.: secondary branches per panicle, SBL: secondary branch length, FGP: filled grains per panicle, UFGP: unfilled grains per panicle, GL: grain length, GB: grain breadth, GLBR: grain length breadth ratio, TGW: 1000-grain weight, YP: yield per plant, and HI: harvest index.

**Table 5:** Estimation of genetic parameters of different quantitative characters in 113 aromatic and fine rice genotypes.

Character	*σ* ^2^_*G*_	*σ* ^2^_*P*_	GCV (%)	PCV (%)	*h*_*B*_^2^ (%)	GA (%)	GA in % of mean
CD (mm)	0.42	0.58	14.03	16.45	72.73	0.87	18.91
FLA (cm^3^)	46.19	63.53	19.10	22.40	72.70	9.16	25.73
DF	56.10	58.36	7.25	7.40	96.13	11.60	11.24
DM	53.30	58.25	5.53	5.79	91.50	11.03	8.36
PH (cm)	199.64	215.76	9.65	10.04	92.53	21.47	14.67
ET No.	1.45	2.60	12.22	16.39	55.56	1.42	14.39
PL (cm)	2.10	3.26	5.06	6.31	64.31	1.83	6.41
PB No.	1.23	2.67	10.72	15.80	46.04	1.19	11.50
PBL (cm)	1.08	1.67	9.66	12.03	64.50	1.32	12.26
SB No.	117.39	130.38	33.17	34.95	90.03	16.24	49.72
SBL (cm)	0.06	0.10	9.24	11.41	65.54	0.33	11.82
FG/P	1384.30	2059.61	24.04	29.32	67.21	48.19	31.14
UFG/P	166.83	408.18	29.77	46.57	40.87	13.05	30.08
GL (mm)	1.79	1.84	18.82	19.07	97.44	2.09	29.36
GB (mm)	0.14	0.15	15.78	16.74	88.84	0.55	23.50
GLBR	0.48	0.51	22.35	23.04	94.16	1.07	34.27
TGW (g)	19.23	20.43	33.18	34.20	94.10	6.72	50.85
YP (g)	2.19	7.55	11.96	22.20	29.03	1.26	10.18
HI	0.00	0.00	13.40	23.21	33.33	0.03	12.22

CD: culm diameter (mm), FLA: flag leaf area, DF: days to flowering, DM: days to maturity, PH: plant height, ET No.: effective tiller number, PL: panicle length, PB No.: primary branches per panicle, PBL: primary branch length, SB No.: secondary branches per panicle, SBL: secondary branch length, FGP: filled grains per panicle, UFGP: unfilled grains per panicle, GL: grain length, GB: grain breadth, GLBR: grain length breadth ratio, TGW = 1000 grain weight, YP: yield per plant, HI: harvest index, *σ* ^2^_*G*_ = genotypic variance, *σ* ^2^_*P*_ = phenotypic variance, (GCV) % = genotypic coefficient of variation,(PCV) % = phenotypic coefficient of variation, *h*_*B*_^2^ (%) = heritability (broad sense), and GA (%) = genetic advance.

**Table 6:** Pearson's correlation coefficient among 19 quantitative traits of aromatic and fine rice genotypes.

	CD	FLA	DF	DM	PL	ET No.	PH	FG	UFG	GL	GB	GLB	PB No.	PBL	SB No.	SBL	TGW	YP	HI
CD	1.00																		
FLA	0.471^*∗∗*^	1.00																	
DF	0.270^*∗∗*^	−0.061	1.00																
DM	0.223^*∗*^	−0.129	0.978^*∗∗*^	1.00															
PL	−0.036	0.004	0.123	0.187^*∗*^	1.00														
ET No.	−0.441^*∗∗*^	−0.242^*∗∗*^	−0.057	−0.029	0.069	1.00													
PH	0.266^*∗∗*^	−0.073	0.268^*∗∗*^	0.306^*∗∗*^	0.265^*∗∗*^	−0.007	1.00												
FG	−0.007	−0.267^*∗∗*^	0.230^*∗*^	0.272^*∗∗*^	0.178	0.012	0.123	1.00											
UFG	−0.103	−0.242^*∗∗*^	−0.143	−0.118	−0.012	0.017	0.062	0.288^*∗∗*^	1.00										
GL	0.153	0.342^*∗∗*^	−0.270^*∗∗*^	−0.334^*∗∗*^	−0.068	−0.012	−0.244^*∗∗*^	−0.595^*∗∗*^	−0.258^*∗∗*^	1.00									
GB	0.152	0.409^*∗∗*^	0.014	0.004	0.204^*∗*^	−0.101	0.064	−0.335^*∗∗*^	−0.018	0.148	1.00								
GLB	0.047	0.048	−0.237^*∗*^	−0.286^*∗∗*^	−0.190^*∗*^	0.049	−0.264^*∗∗*^	−0.312^*∗∗*^	−0.239^*∗*^	0.763^*∗∗*^	−0.511^*∗∗*^	1.00							
PB No.	0.413^*∗∗*^	0.115	0.168	0.160	−0.140	−0.231^*∗*^	0.135	0.081	0.053	−0.060	−0.020	−0.055	1.00						
PBL	−0.285^*∗∗*^	0.085	−0.204^*∗*^	−0.171	0.359^*∗∗*^	0.243^*∗∗*^	−0.229^*∗*^	0.218^*∗*^	−0.071	0.039	−0.043	0.083	−0.383^*∗∗*^	1.00					
SB No.	−0.116	−0.218^*∗*^	0.038	0.075	0.107	0.044	−0.013	0.784^*∗∗*^	0.351^*∗∗*^	−0.534^*∗∗*^	−0.301^*∗∗*^	−0.267^*∗∗*^	0.011	0.345^*∗∗*^	1.00				
SBL	−0.109	0.125	−0.299^*∗∗*^	−0.316^*∗∗*^	0.093	0.268^*∗∗*^	−0.198^*∗*^	0.115	0.032	0.286^*∗∗*^	−0.087	0.295^*∗∗*^	−0.319^*∗∗*^	0.598^*∗∗*^	0.283^*∗∗*^	1.00			
TGW	0.108	0.479^*∗∗*^	−0.041	−0.058	0.200^*∗*^	−0.066	−0.154	−0.491^*∗∗*^	−0.421^*∗∗*^	0.651^*∗∗*^	0.501^*∗∗*^	0.249^*∗∗*^	−0.245^*∗∗*^	0.266^*∗∗*^	−0.445^*∗∗*^	0.250^*∗∗*^	1.00		
YP	0.143	0.042	0.407^*∗∗*^	0.431^*∗∗*^	0.190^*∗*^	0.118	−0.023	0.267^*∗∗*^	−0.141	−0.001	0.016	−0.003	−0.073	0.324^*∗∗*^	0.231^*∗*^	0.324^*∗∗*^	0.258^*∗∗*^	1.00	
HI	−0.140	−0.152	0.025	0.043	0.056	0.048	−0.216^*∗*^	0.343^*∗∗*^	0.008	−0.194^*∗*^	−0.282^*∗∗*^	0.034	−0.120	0.390^*∗∗*^	0.436^*∗∗*^	0.324^*∗∗*^	−0.112	0.545^*∗∗*^	1.00

*∗∗* and *∗* indicate significance at 1% and 5% level of probability, respectively.

CD: culm diameter (mm), FLA: flag leaf area, DF: days to flowering, DM: days to maturity, PL: panicle length, ET No.: effective tiller number, PH: plant height, FGP: filled grains per panicle, UFGP: unfilled grains per panicle, GL: grain length, GB: grain breadth, GLBR: grain length breadth ratio, PB No.: primary branches per panicle, PBL: primary branch length, SB No.: secondary branches per panicle, SBL: secondary branch length, TGW: 1000-grain weight, YP: yield per plant, and HI: harvest index.

**Table 7:** Latent roots (eigen values) and their variation in 19 quantitative characters in 113 aromatic and fine rice genotypes.

Principal component axes	Latent roots	Variation (%)	Cumulative% of variation
I	2.731	13	48.8
II	2.178	10.37	59.17
III	1.613	7.68	66.85
IV	1.219	5.81	72.66
V	1.01	4.81	77.47
VI	0.791	3.76	81.23
VII	0.669	3.19	84.42
VIII	0.654	3.12	87.54
IX	0.551	2.62	90.16
X	0.476	2.26	92.42
XI	0.372	1.77	94.19
XII	0.293	1.4	95.59
XIII	0.258	1.23	96.82
XIV	0.22	1.05	97.87
XV	0.176	0.84	98.71
XVI	0.151	0.72	99.43
XVII	0.096	0.46	99.89
XVIII	0.014	0.08	99.97
XIX	0.007	0.03	100

**Table 8:** Distribution of 113 aromatic and fine rice genotypes into ten clusters.

Cluster	Number of genotypes	% total	Name of genotypes
I	19	16.81	Nunia, Chini Sagar (2), Tilkapur, Kalobhog, Jabsiri, Chinisakkor, Noyonmoni, Tulsimoni, Khirshabuti, Gua masuri, Rajbhog, Baoijhaki, Tulsimala, Desikatari, Thakurbhog, Tulsimaloty, Radunipagal, Khasa, and Kataribhog
II	11	9.73	Begun bichi, Elai, Bashmati 370, Sugandhi dhan, Khazar, Basmati Sufaid 106, BRRI dhan50, Basmati 37, Basnatu sufaid 187, BU dhan2R, and Bashful
III	17	15.04	Jirabuti, Soru kamina, Kamini soru, Doiarguru, Luina, Kalijira (short grain), Philliphine kataribhog, Jirabhog (Bolder), Uknimodhu, Jira dhan, Badshabhog, Kalijira (long grain), Jesso balam, Dakshahi, Straw, Dubsail, and Sunduri samba
IV	13	11.50	Sagardana, Kalgochi, Chiniatob, Gopalbhog, Hatisail, Buchi, BRRI dhan38, Gordoi, Basmati, Padmabhog, Oval TAPL-2990, Kalijira TAPL-74, and Kalobakri
V	8	7.08	Saubail, Begunmala, Rajbut, Ranisalut, Gandhakusturi, Jirakatari, Awned TAPL-545, and Black TAPL-554
VI	3	2.65	Sakkorkhana, Kalijira TAPL-64, and Kalijira TAPL-68
VII	6	5.31	Chini Kanai, Chinigura, Khaskani, BRRI dhan34, BRRI dhan37, and Chinisail
VIII	18	15.92	Chinniguri, Premful, Baoibhog, Sakkorkhora, BR5, Uknimodhu, Chiniatob-2, Begunbichi-2, Bhatir cikon, Dolagocha, Dhan chikon, Badshabhog-2, Malshira, Sadagura, Chinikanai, Meedhan, Gobindhabhog, and Fulkari
IX	8	7.08	Sakor, Binnaphul, Lal Soru, Duksail, Tilokkachari, Chinairri, Kalonunia, and Thakurbhog-2
X	10	8.85	Meny, Kalomala, Khasa Mukpura, Bawaibhog-2, Khuti chikon, Tulsimala-2, Modhumadab, Parbatjira, Dudsail, and Maloti

**Table 9:** Ten higher and ten lower intergenotypic distances among the 113 aromatic and fine rice genotypes.

Sl. No.	Genotypic combination	Distances
(a) Ten higher intergenotypic distance		
01	Gopalbhog-Kalobakri	2.274
02	Haitsail TAPL101-Kalobakri	2.126
03	Buchi-BU dhan 2R	1.836
04	Kalgochi-Kutichikon	1.832
05	Begun Mala-Elai	1.779
06	BRRI dhan50-Bashful	1.632
07	Elai-Khazar	1.589
08	Khasa Mukpura-Dudsail	1.580
09	Ranisalut-Jirakatari	1.528
10	Jirabuti-Straw TAPL-554	1.522

(b) Ten lower intergenotypic distance		
01	Doiagura-Jiradhan	0.299
02	Kamini soru-Jirabhog (bolder)	0.301
03	Kutichikon-Parbatjira	0.318
04	Chinisagor (2)-Khasa	0.321
05	Tilkapur-Guamasuri	0.333
06	Kalobhog-Noyonmoni	0.341
07	Noyonmoni-Rajbhog	0.356
08	Chinisagor (2)-Jabsiri	0.361
09	Jabsiri-Chinisakkor	0.362
10	Tilkapur-Kalobhog	0.374

**Table 10:** Average intra- (bold) and intercluster distances (*D* ^2^) for 113 aromatic and fine rice genotypes.

Cluster	I	II	III	IV	V	VI	VII	VIII	IX	X
I	**0.68**	7.499	3.710	4.274	4.807	7.332	3.975	7.592	4.895	12.119
II		**1.27**	9.438	7.161	8.645	10.77	9.681	12.206	9.087	15.791
III			**0.65**	6.288	7.713	6.876	4.332	4.799	3.775	9.087
IV				**1.01**	4.46	6.946	5.365	8.983	4.908	13.609
V					**0.96**	9.198	6.713	11.435	7.951	16.116
VI						**0.61**	7.524	7.896	6.498	10.93
VII							**0.75**	6.461	5.036	11.064
VIII								**0.71**	4.78	5.294
IX									**0.79**	9.256
X										**0.90**

**Table 11:** Cluster means for 19 quantitative characters in 113 aromatic and fine rice genotypes.

	I	II	III	IV	V	VI	VII	VIII	IX	X
CD (mm)	4.57	4.75	4.97	4.53	4.61	**5.86 (H)**	4.42	**4.16 (L**)	4.2	5.08
FLA (cm^3^)	33.37	**43.25 (H)**	35.12	35.77	36.82	40.16	33.45	33.89	**30.95 (L)**	34.2
DF	101	**90.33 (L)**	102.5	107.56	106.75	**120.11 (H)**	107.8	104.7	103.7	104.03
DM	130.1	**118.2 (L)**	131.1	136.13	135.12	**146.67 (H)**	135.8	133.7	132.9	133.07
PH (cm)	152	**125.2 (L)**	158.4	138.5	157.56	**168 (H)**	131.2	143.7	142.1	146.39
ET No.	9.99	9.15	**9.08 (L)**	**10.44 (H)**	10.33	9.19	9.92	10.21	9.96	9.8
PL (cm)	28.67	27.57	28.56	28.99	28.81	27.95	**27.4 (L)**	28.98	28.85	**29.28 (H)**
PB No.	10.67	**9.80 (L)**	**10.85 (H)**	10.15	9.82	11.22	10.58	9.83	10.61	10.4
PBL (cm)	10.25	11.28	**10.11 (L)**	10.85	10.26	10.15	10.45	**11.42 (H)**	11.02	11.40
SB No.	28.38	22.96	35.44	23.83	**18.97 (L)**	25.78	37.56	42.53	34.06	**49.5 (H)**
SBL (cm)	2.72	**3.03 (H)**	2.65	2.61	**2.60 (L)**	2.58	2.74	2.83	2.65	2.99
FG/P	135.5	**98.84 (L)**	162.9	125.96	102.69	158.11	156.3	198.3	162.2	**232.2 (H)**
UFG/P	53.41	28.3	51.78	27.46	43.45	**13.89 (L)**	**65.44 (H)**	47.91	28.91	46.31
GL (mm)	7.36	**9.67 (H)**	6.81	7.26	7.71	6.34	6.57	**6.13 (L)**	6.61	6.32
GB (mm)	2.31	2.33	2.36	**2.42 (H)**	2.84	**1.89 (L)**	2.36	2.21	2.34	2.13
GLBR	3.21	**4.28 (H)**	2.91	3.12	2.81	3.36	2.8	**2.79 (L)**	2.91	3.0
TGW (g)	11.83	**19.03 (H)**	11.59	15.49	17.46	12.28	**11.02 (L)**	11.16	13.17	11.25
YP (g)	**11.02 (L)**	11.59	11.96	13.29	11.78	**14.06 (H)**	13.94	12.77	12.42	13.60
HI	0.22	0.22	0.23	0.24	**0.21 (L**)	0.23	0.26	0.25	0.24	**0.27 (H)**

CD: culm diameter (mm), FLA: flag leaf area, DF: days to flowering, DM: days to maturity, PH: plant height (cm), ET No.: effective tiller number, PL: panicle length (cm), PB No.: primary branches per panicle, PBL: primary branch length (cm), SB No.: secondary branches per panicle, SBL: secondary branch length (cm), FGP: filled grains per panicle, UFGP: unfilled grains per panicle, GL: grain length (mm), GB: grain breadth (mm), GLBR: grain length breadth ratio, TGW: 1000-grain weight (g), YP: yield per plant (g), and HI: harvest index.
